# Multi-omic analyses of the same sample using metabolomics, lipidomics, proteomics, phosphoproteomics, and glycoproteomics

**DOI:** 10.1039/d6cc03477b

**Published:** 2026-08-10

**Authors:** Yuefan Wang, Xuejun Peng, Hongyi Liu, Mia Gidley, Beth Kelly, Erika L. Pearce, Edward J. Pearce, Xianlin Han, Hui Zhang

**Affiliations:** a Department of Pathology, Johns Hopkins University Baltimore Maryland 21231 USA ywang298@jhmi.edu; b Bruker Scientific, San Jose California 95134 USA; c Bloomberg-Kimmel Institute for Cancer Immunotherapy, Department of Oncology, Johns Hopkins University School of Medicine Baltimore Maryland 21287 USA; d Sam and Ann Barshop Institute for Longevity and Aging Studies, University of Texas Health Science Center San Antonio TX 78299 USA; e Department of Medicine, University of Texas Health Science Center San Antonio San Antonio TX 78299 USA

## Abstract

Mass spectrometry (MS)-based multi-omics offers powerful tools to comprehensively characterize proteins, post-translational modifications, metabolites, and lipids. However, these measurements are typically performed using separate sample preparation workflows and modality-specific liquid chromatography mass spectrometry (LC–MS) platforms, limiting integration and constraining applications to small amounts of sample materials, especially scarce clinical specimens. Here, we describe a unified nano-LC–MS framework that enables metabolomic, lipidomic, proteomic, phosphoproteomic, and glycoproteomic analyses from the same starting material using a single nano-LC–MS platform, with only the chromatographic conditions, acquisition methods, and enrichment procedures tailored to each omics. This integrated strategy reduces workflow complexity and sample consumption while improves analytical continuity across molecular layers. By enabling deep multi-omics characterization from the same sample, this platform provides a practical foundation for comprehensive analysis of precious clinical samples.

## Abstract 2

An LC/MS-based deep multi-omics platform for integrated molecular characterization from a single sample.

## Graphical abstract

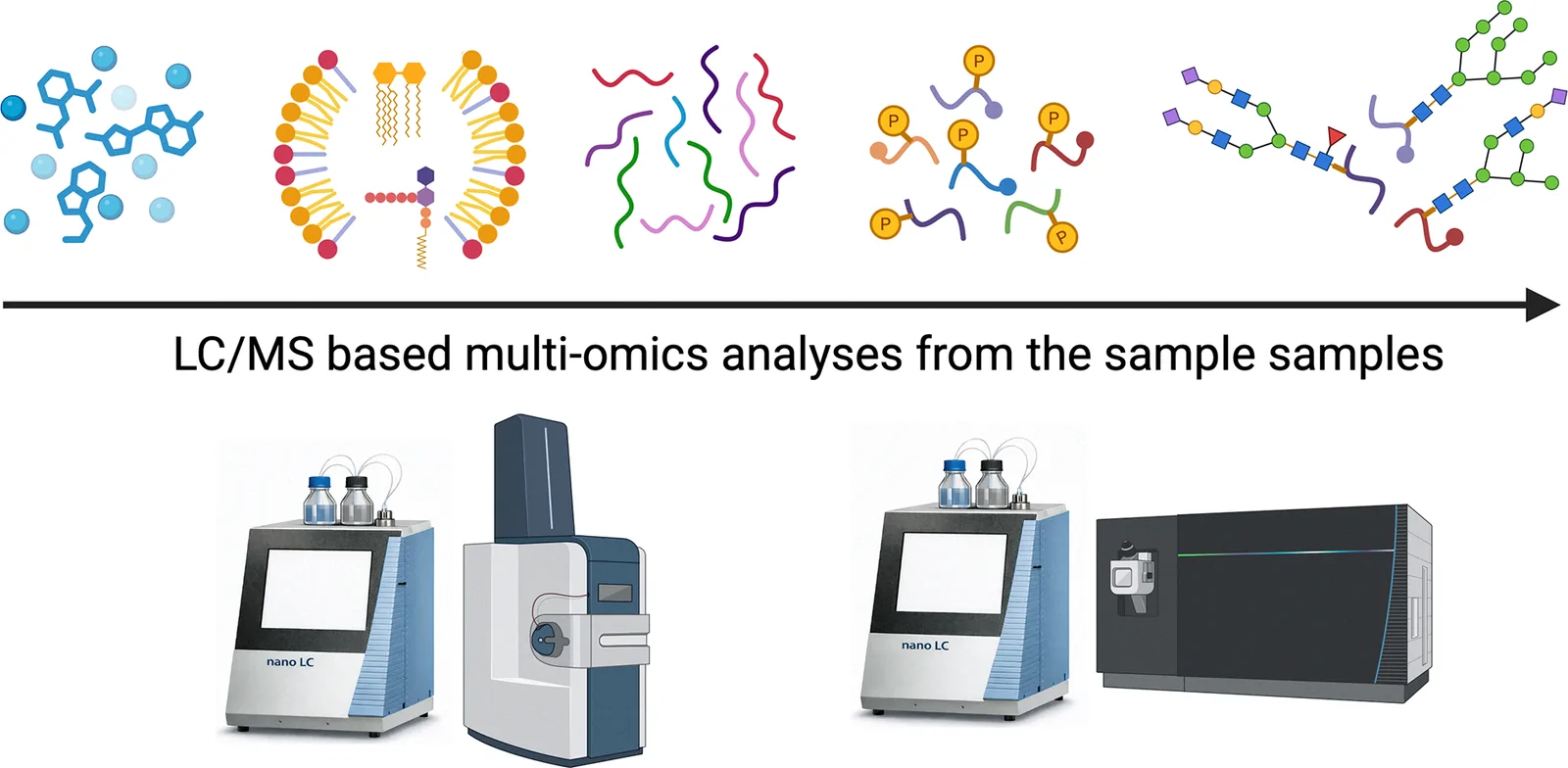

The proteome represents the functional output of gene expression,^1,2^ whereas protein modifications such as phosphorylation and glycosylation, as well as the small molecular products of functional proteomes such as metabolites and lipids, capture downstream biochemical and phenotypic states.^3–5^ These molecular layers provide a complementary and integrated view of biological systems and form an essential foundation for data-driven precision medicine.^6–9^ In particular, coordinated multi-omic analysis of proteins, metabolites, and lipids has the potential to improve understanding of disease mechanisms at molecular levels and support precision medicine.^2,6,7,9^

Mass spectrometry (MS) has emerged as a powerful platform for comprehensive characterization of these molecular classes because of its sensitivity, versatility, and broad analytical coverage.^10–12,41^ However, current MS-based multi-omics profiling workflows still typically rely on separate sample preparation procedures and distinct liquid chromatography and mass spectrometry (LC–MS) platforms for different molecular classes,^13^ which increases workflow complexity and higher sample consumption. These limitations are particularly challenging for limited specimens, where the available material is often limited to support multiple parallel workflows. This is especially important for clinical samples, which could often be the only material available for personalized diagnosis, molecular profiling, and treatment decision-making. There is an urgent need to develop multi-omics workflows that use the same specimen within a unified nano-LC–MS (nLC–MS) system while enabling deep profiling across these biomolecular classes.

Proteomics analyses are typically performed on nLC–MS platforms,^14^ whereas metabolites and lipids (referred to as small biomolecules) are more commonly characterized using analytical-LC–MS systems (aLC–MS).^11^ This technical difference contributes to the separation of proteomics, metabolomics, and lipidomics into largely independent analytical workflows, each with distinct chromatographic conditions, instrumentation, and sample handling requirements. Recent studies have begun to show that nLC–MS can also support small biomolecule analysis with improved sensitivity,^15–17^ suggesting the possibility of integrating these molecular measurements within a common nLC–MS centered workflow. In parallel, simultaneous extraction of multiple biomolecular classes has also been reported,^18^ establishing the feasibility of multimolecular analysis from a single specimen. Nevertheless, these studies generally continue to rely on separate downstream analytical platforms and have not been primarily designed to maximize deep molecular characterization across all biomolecular layers.

Building on these advances,^15–18^ we developed a workflow for the integrated analysis of metabolites, lipids, proteins, phosphoproteins, and glycoproteins within a unified nLC–MS analytical framework. To achieve broad and deep molecular coverage, modality-optimized MS configurations were used for individual molecular classes while maintaining a common workflow concept and shared starting material. We show that extraction of small biomolecules prior to proteomic sample processing allows the integrated multi-omic analyses of metabolomics, lipidomics, and proteomics, demonstrating strong concordance with conventional proteomic analyses performed without prior extraction. Importantly, phosphopeptide and intact glycopeptide profiling were likewise preserved. These results establish a practical and robust strategy for comprehensive multi-omics characterization from the same specimen, with value for limited and precious clinical samples.

To achieve comprehensive molecular profiling from a single specimen, it is critical to develop a coordinated extraction strategy that harmonizes the divergent chemical requirements of different omics layers. Different classes of small biomolecules require distinct organic solvent combinations for efficient extraction from samples.^19,20^ In contrast, protein lysis buffers often contain protease, phosphatase, and glycosidase or glycosylation-related enzyme inhibitors,^13,21^ many of which may also dissolve into organic solvents during small biomolecule extraction. To minimize interference of metabolite and lipid extraction to the downstream proteomic workflows and preserve protein integrity, metabolite and lipid extraction were therefore performed prior to protein extraction, forming the basis of our multi-omics workflow (Fig. 1). A patient-derived xenograft (PDX) sample was utilized as a reference sample.^22,23^ In the first step, metabolites and environmentally-derived exposome molecules were extracted from a cryopulverized tissue specimen using methanol.^13^ Lipids and lipid-like compounds were subsequently extracted using an isopropanol/water (8 : 1, v/v) solution.^24^ The remaining sample pellet was then lysed in lysis buffer and sonicated three times for 20 seconds each.^23^ Following reduction, alkylation, and tryptic digestion, peptides were purified and subjected to proteomic analysis.^21^ Phosphopeptides were subsequently enriched from the global peptide pool using an immobilized metal affinity chromatography (IMAC)-based workflow,^21^ and the corresponding flow-through was further processed on a mixed-mode strong anion exchange (MAX) column to enrich intact glycopeptides.^25,26^ In parallel, we also generated proteomic, phosphoproteomic, and glycoproteomic datasets using the same sample but without prior metabolite and lipid extraction.

**Fig. 1:**
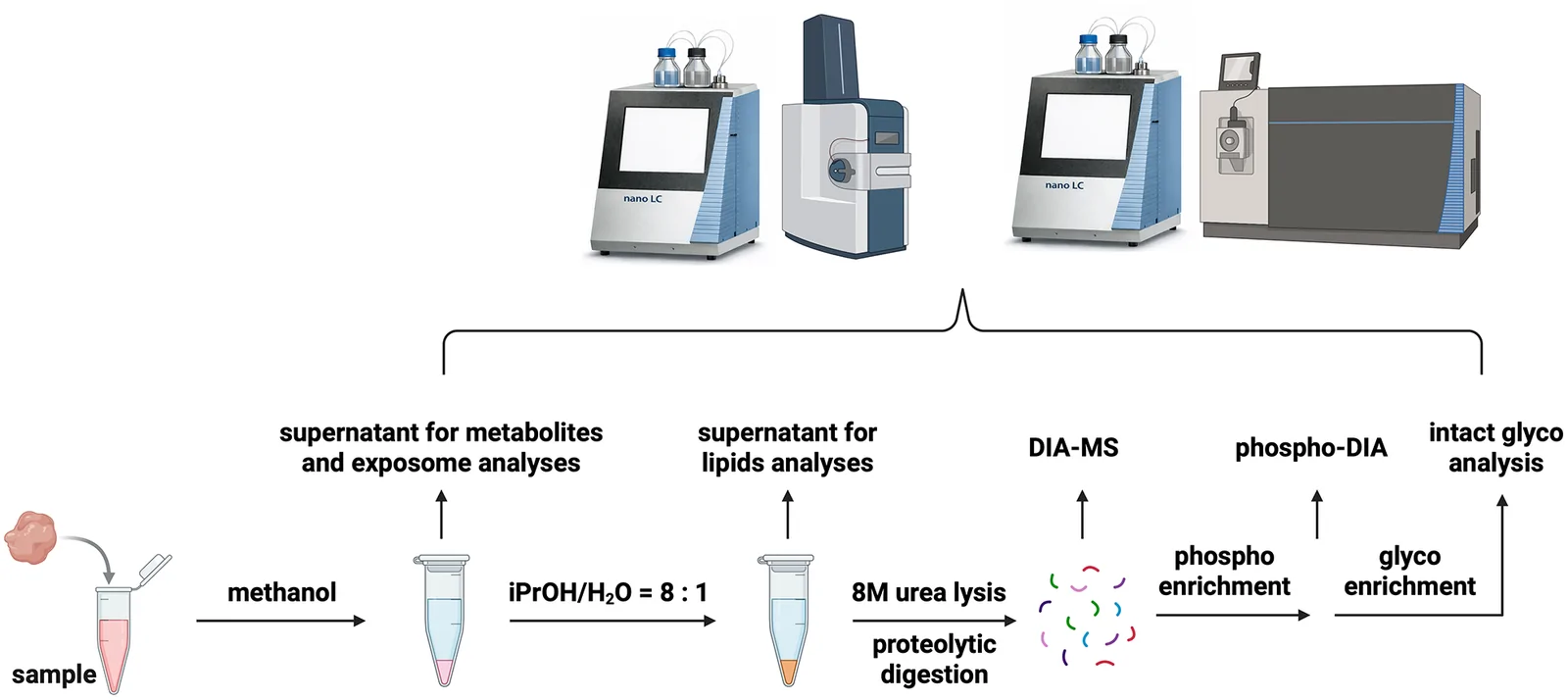
Multi-omics profiling workflow. Metabolites, lipids, proteins, phosphopeptides, and intact glycopeptides are sequentially extracted and analyzed from the same specimen within a unified nLC–MS instrument combination framework.

Together, these considerations point to the central question in sequential same-sample multi-omics workflows: whether extraction of metabolites and lipids can be achieved without sacrificing downstream proteome, phosphoproteome, and intact glycopeptide characterization.

To validate the efficacy of our sequential extraction and profiling strategy, we first performed a characterization of the metabolic and lipidomic landscapes captured by the nLC–MS workflow. We first generated metabolomic and lipidomic datasets in both positive and negative ion acquisition modes, with three replicates for each mode in nLC–MS platform. Small biomolecule features consistently quantified across all replicates were retained for downstream annotation and comparison. Using MetaboScape-generated data matrices,^27,28^ we annotated features against the Human Metabolome Database (HMDB)^29,30^ for metabolite assignment and LIPID MAPS^31,32^ for lipid annotation. A broad range of small biomolecule features were identified in the methanol-extracted fraction. Most annotated features in this fraction were metabolites, including compounds involved in biosynthesis of unsaturated fatty acids, linoleic acid metabolism, as well as pantothenate and CoA biosynthesis by MetaboAnalyst KEGG pathway enrichment^30^ (Fig. S1a). The methanol fraction also contained a smaller subset of lipid-related molecules, such as lysophosphatidylcholines (LPCs or LysoPCs) and alkylacylglycerophosphocholines [PC(O−)], together with a limited number of exogenous compounds, including ampicillin (Table S1). In contrast, the isopropanol-extracted fraction was enriched predominantly for diverse lipid classes, with fewer polar metabolites and exogenous molecules detected (Table S2). These results demonstrate that the two extraction fractions provide complementary coverage of polar metabolites and lipids in samples.

To further assess analytical performance, we compared our nLC–MS configuration against the conventional analytical-scale LC–MS (aLC–MS) platform using timsTOF. In positive ion acquisition mode, 152 small biomolecules were commonly detected in both workflows (Table S3), providing a shared set of features for direct evaluation of the two LC configurations. Pathway enrichment analysis of these commonly detected features revealed substantial overlap with the pathways identified by nLC–MS alone, including biosynthesis of unsaturated fatty acids and linoleic acid metabolism (Fig. S1b). Together, these results indicate that nLC–MS captures biologically relevant small biomolecule signatures comparable to those obtained by aLC–MS, while supporting robust metabolite profiling on the timsTOF MS platform.

Among the annotated lipid features, we identified 113 lipids reported in recent publications,^28,33–35^ together with an additional 173 lipids matched to SwissLipids IDs,^36^ giving a total of 286 annotated lipids in the isopropanol-extracted fraction (Table S2). These lipids were distributed across 6 main lipid categories and 41 class types (Table 1). Glycerophospholipids represented the largest group, comprising 173 lipids across 27 class types, with ether phosphatidylcholines being prominently represented. Sphingolipids were the second most abundant category, with 57 lipids across 4 class types, including ceramides and sphingomyelins, followed by glycerolipids with 31 lipids across 4 class types, mainly diacylglycerols. Smaller subsets included sterol lipids (12 lipids), phosphoinositides (7 lipids), and fatty acyls (6 lipids). The broad coverage of these diverse lipid species, particularly complex membrane lipids, underscores the suitability of the nLC–MS workflow for in-depth lipidomics within a multi-omics context.

**Table 1:** Summary of annotated lipid classes identified in the isopropanol-extracted fraction (replicate *n* = 3)

Main class	Class type	Major class	Total lipids
Fatty acyls	2	Free fatty acid	6
Glycerolipids	4	Diacylglycerol	31
Glycero-phospholipids	27	Ether phosphatidylcholine	173
Phospho-inositides	2	Phosphatidylinositol bisphosphate	7
Sphingolipids	4	Ceramide and sphingomyelin	57
Sterol lipids	2	Sterol ester	12

We next evaluated the nLC–timsTOF MS workflow to establish a robust analytical basis for reproducible metabolite and lipid detection in sample extracts.

We assessed the reproducibility of the metabolite-enriched fraction by calculating coefficients of variation (CVs) for commonly quantified small-molecule features. The metabolomics dataset showed high technical precision, with a median CV of 11.0%, and more than 80% of annotated features displaying CVs below 20% (Fig. 2a). We then examined the lipid-enriched fraction, focusing on commonly quantified lipid-related features. Compared with the metabolite fraction, the lipidomics dataset showed slightly greater variability but remained reproducible overall, with a median CV of 12.9% and more than 76% of annotated features falling below CV of 20% (Fig. 2b). Collectively, these findings demonstrate that the extraction workflow provides stable and reproducible detection across both metabolite- and lipid-enriched fractions, supporting its use in integrated downstream multi-omics analyses.

**Fig. 2:**
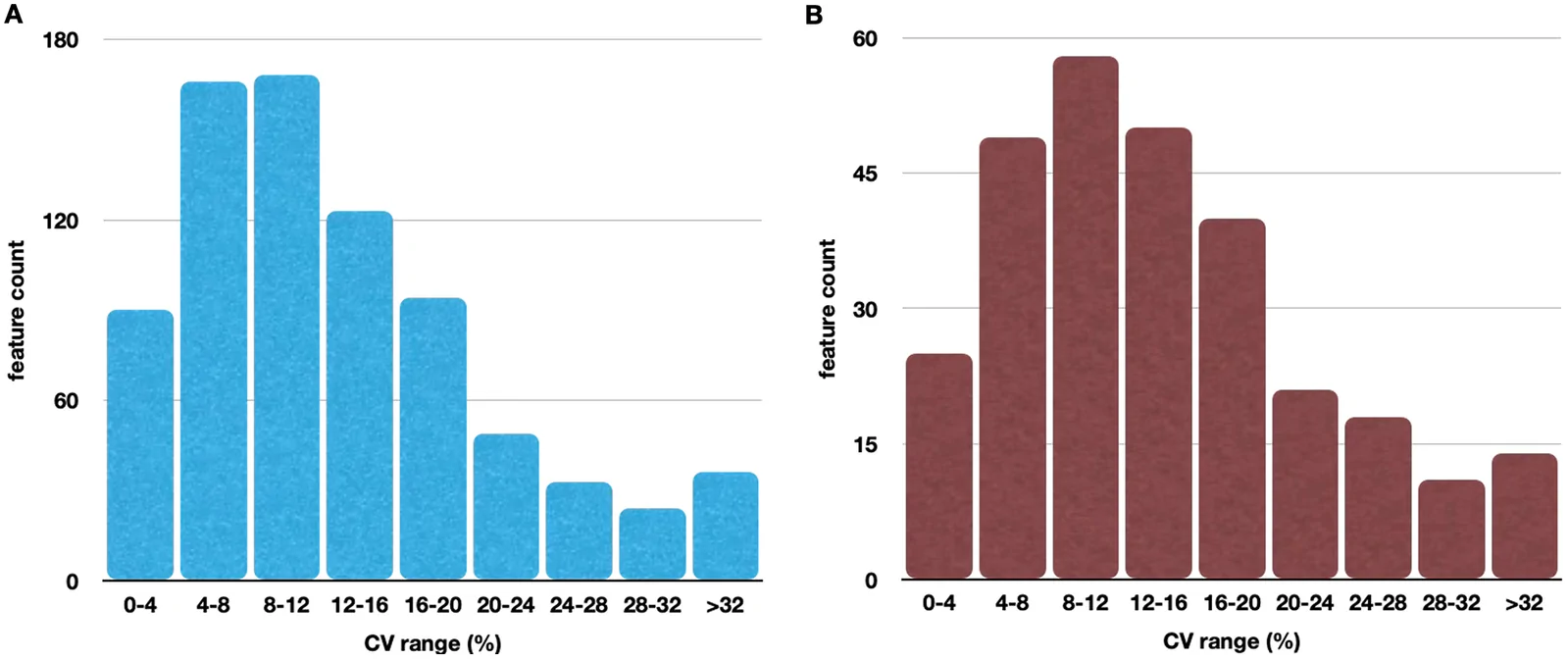
Reproducibility of small-biomolecule profiling by nLC–MS (replicate *n* = 3). (A) Distribution of CVs for features detected in the metabolite-enriched fraction. (B) Distribution of CVs for features detected in the lipid-enriched fraction.

To determine whether prior extraction of metabolites and lipids sacrifices downstream protein-based analyses, we compared proteomic, phosphoproteomic, and glycoproteomic datasets generated with and without small biomolecule extraction (referred to as “with and without extraction”). This comparison allowed us to determine whether removal of small biomolecules affects proteome and PTM coverage as well as quantitative agreements.

Proteome and phosphoproteome characterizations were performed using data-independent acquisition mass spectrometry (DIA-MS),^37–39^ while glycoproteome characterization was performed using data-dependent acquisition mass spectrometry (DDA-MS).^40^ In the global proteomic analysis (Table S4), the number of quantified proteins was very close (11 134 *vs.* 11 073) with and without prior small biomolecule extraction and 11 036 protein groups were commonly quantified in the two platforms (Fig. 3a), demonstrating that the overall proteome was largely retained across the two workflows. Quantitative comparison showed a pairwise Spearman correlation coefficient of 0.988 (Fig. 3b), indicating strong agreement and minimal impact of prior metabolite and lipid extraction on proteome profiling. Consistent with this result, phosphoproteomic analysis identified 23 679 and 23 050 localized phosphosites that were quantified in each workflow respectively (Fig. 3c), with approximately 80% overlap at the phosphopeptide level (Table S5). The pairwise Spearman correlation coefficient of phosphopeptide quantification was 0.945 (Fig. 3d), further supporting that prior small biomolecule extraction did not substantially compromise downstream phosphoproteome analysis. In the glycoproteomic profiles between the two workflows^25,40^ (Table S6), there were 685 and 667 intact glycopeptides identified in each pipeline respectively (Fig. 3e), and glycopeptide quantification showed a pairwise Spearman correlation coefficient of 0.911 (Fig. 3f). These results indicate that prior small biomolecule extraction did not substantially affect downstream intact glycopeptide detection or quantitative analysis.

**Fig. 3:**
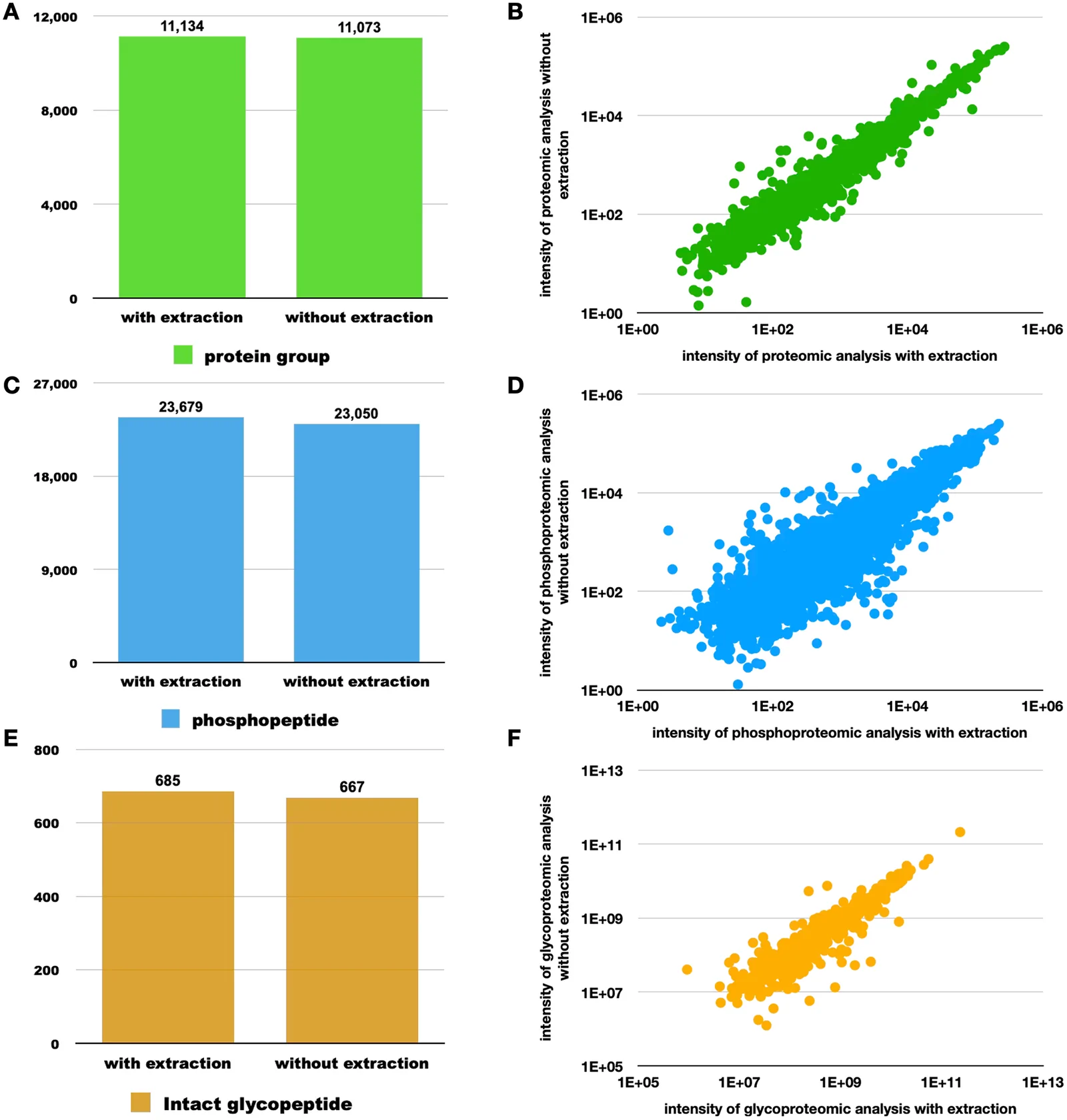
Comparison of proteomic, phosphoproteomic, and glycoproteomic analyses with and without prior small biomolecule extraction. (A) Protein identifications with and without prior extraction. (B) Pairwise Spearman correlation of protein intensities with and without prior extraction. (C) Phosphopeptide identifications with and without prior extraction. (D) Pairwise Spearman correlation of phosphopeptide intensities with and without prior extraction. (E) Intact glycopeptide identifications with and without prior extraction. (F) Pairwise Spearman correlation of intact glycopeptide intensities with and without prior extraction.

Overall, these results showed that the sequential workflow maintained strong analytical compatibility across proteome, phosphoproteome, and intact glycoproteome measurements after prior metabolite and lipid extraction.

Proteomics, metabolomics, and lipidomics provide unique and complementary information for understanding the functional outputs of gene expression and the physiological/pathological state of tissues.^41^ Yet these molecular layers are commonly analyzed using separate platforms and independent sample preparation workflows, increasing both sample requirements and experimental complexity. Here, we developed a sequential multi-omics pipeline for stepwise profiling of metabolites, lipids, proteins, phosphopeptides, and intact glycopeptides from the same specimen. We showed that prior extraction of small biomolecules with methanol and isopropanol did not adversely affect downstream proteome or PTM characterizations. Protein, phosphopeptide, and intact glycopeptide identifications were highly similar across workflows, and pairwise intensity correlations between workflows with and without prior extraction exceeded 0.90. Moreover, all datasets were generated using nLC–MS, establishing a unified and sample-sparing platform for integrated multi-omics. In summary, this study supports the feasibility of sequential multi-omics profiling from same samples and provides a robust framework for comprehensive molecular analysis in future biological and clinical studies.

## Conflicts of interest

There are no conflicts to declare.

## Supplementary Material

CC-062-D6CC03477B-s001

CC-062-D6CC03477B-s002

## Data Availability

All datasets generated in this study, detailed materials and methods, and supplementary figure are included in the supplementary information (SI). Supplementary information is available. See DOI: https://doi.org/10.1039/d6cc03477b.
