# Supplementary material for: Multi-omic analyses of the same sample using metabolomics, lipidomics, proteomics, phosphoproteomics, and glycoproteomics
Source: Chem Commun (Camb). 2026 Aug 10;62(72):17948–52. doi: 10.1039/d6cc03477b (PMC13491391; doi:10.1039/d6cc03477b)
Supplement: Supplementary file 1 [file CC-062-D6CC03477B-s001.pdf]

Supplementary Information (SI) for ChemComm.
This journal is © The Royal Society of Chemistry 2026

Supplementary Information (SI): Multi-Omic Analyses of the Same Sample using Metabolomics, Lipidomics, Proteomics, Phosphoproteomics, and Glycoproteomics

Yuefan Wang,[a,*] Xuejun Peng,[b] Hongyi Liu,[a] Mia Gidley,[c] Beth Kelly,[c] Erika L. Pearce,[c] Edward J. Pearce,[c] Xianlin Han,[d] and Hui Zhang,[a]

a. Department of Pathology, Johns Hopkins University, Baltimore, Maryland 21231, United States.

b. Bruker Scientific, San Jose, California 95134, United States.

c. Bloomberg-Kimmel Institute for Cancer Immunotherapy, Department of Oncology, Johns Hopkins University School of Medicine, Baltimore, Maryland 21287, United States.

d. Sam and Ann Barshop Institute for Longevity and Aging Studies, University of Texas Health Science Center, San Antonio, TX, 78299, USA; Department of Medicine, University of Texas Health Science Center San Antonio, San Antonio, TX, 78299, USA

Materials and Methods

Small Biomolecule Extraction

For metabolite extraction, 0.5 mL methanol/water solution (MeOH/H_2O, 4:1, v/v) was added to 100 μg sample. The mixture was vortexed three times for 30 s each and then incubated on ice for 30 min. After incubation, the extract was centrifuged at 12,000 rpm for 10 min at 4°C, and the supernatant was transferred to a new tube. The remaining sample pellet was extracted again with 0.4 mL of MeOH/H_2O solution (4:1, v/v), incubated on ice for an additional 10 min, and centrifuged at 16,000 rpm for 10 min at 4°C. The two supernatants were combined and dried using a SpeedVac. The dried metabolite extracts were stored at −80°C until further use or reconstituted in 500 μL of 50:50 acetonitrile/water for LC-MS analysis. Injection volumes were 2 μL for analytical LC-MS and 1 μL for nanoLC-MS.

For lipid extraction, the remaining sample pellet after metabolite extraction was resuspended in 100 μL water and mixed for 5 min at 1,400 rpm. Subsequently, 800 μL isopropanol was added, and the mixture was incubated with mixing at 2–8°C for 2 h. Samples were then centrifuged at 10,000 rpm for 10 min at 4°C, and the resulting extracts were dried using a SpeedVac. Dried lipid extracts were reconstituted in 900 μL chloroform/isopropanol/acetonitrile/water solution at a ratio of 20:65:35:5 (v/v/v/v). Injection volumes were 1 μL for nanoLC-MS.

Protein Extraction and Digestion for Global Proteomic Analysis

For protein extraction, the sample pellet remaining after small-molecule enrichment, or samples not subjected to small-molecule enrichment, was lysed in 400 μL of 8 M urea lysis buffer containing 8 M urea, 75 mM NaCl, 50 mM Tris-HCl pH 8.0, 1 mM EDTA, 2 μg/mL aprotinin, 10 μg/mL leupeptin, 1 mM PMSF, 10 mM NaF, phosphatase inhibitor cocktail 2, phosphatase inhibitor cocktail 3, and 20 μM PUGNAc. Phosphatase inhibitor cocktails 2 and 3 were each used at a 1:100 dilution. Samples were vortexed three times for 30 s each and sonicated on ice-water for 30 s three times. Insoluble debris was removed by centrifugation at 16,000 × g for 15 min at 4°C. The protein-containing supernatant was collected, and protein concentration was determined using a BCA assay. The lysate was then adjusted to 8 mg/mL with 8 M urea lysis buffer.

Proteins (1.0 mg) were reduced with 6 mM dithiothreitol at 37°C for 1 h and alkylated with 12 mM iodoacetamide at room temperature in the dark for 45 min. After alkylation, the urea concentration was diluted to 2 M using 50 mM Tris-HCl buffer, pH 8.0. Proteins were sequentially digested with Lys-C at an enzyme-to-protein ratio of 1 mAU:50 mg for 2 h at room temperature, followed by trypsin digestion at an enzyme-to-protein ratio of 1 mg:50 mg overnight at room temperature. Proteolytic reactions were quenched by adding 50% formic acid to adjust the pH to <3.

For global proteomic analysis, approximately 1 μg peptides were loaded onto Evotips according to the Evosep One protocol and analyzed using a timsTOF HT mass spectrometer. The remaining digested peptides were desalted using reversed-phase C18 columns and dried using a SpeedVac concentrator for downstream enrichment analyses.

Phosphopeptide Enrichment

Phosphopeptide enrichment was performed using immobilized metal affinity chromatography (IMAC). Briefly, Ni^{2+}-NTA agarose beads were conditioned and incubated with 10 mM $FeCl_3$ aqueous solution at

room temperature for 1 h to generate Fe^{3+}-NTA agarose beads. Peptides from each condition were dissolved in 80% acetonitrile containing 0.1% trifluoroacetic acid (TFA) and incubated with 10 µL of Fe^{3+}-IMAC beads for 30 min.

After incubation, samples were centrifuged at 1,000 × g for 1 min, and the supernatant containing unbound peptides was collected. The beads were resuspended in 80% acetonitrile containing 0.1% TFA and loaded onto conditioned C18 StageTips. The StageTips were washed sequentially with 80% acetonitrile containing 0.1% TFA and 1% formic acid. The flow-through fractions were collected and combined with the unbound peptides from the IMAC enrichment for subsequent glycopeptide enrichment.

Phosphopeptides were eluted from the Fe^{3+}-IMAC beads onto conditioned C18 StageTips using 70 µL 500 mM dibasic potassium phosphate, pH 7.0, three times. The StageTips were then washed twice with 1% formic acid, and phosphopeptides were eluted twice with 50% acetonitrile containing 0.1% formic acid. The enriched phosphopeptides were dried and reconstituted in 3% acetonitrile containing 0.1% formic acid for about 2.5 injection amount. For LC-MS/MS analysis, one injection of the phosphopeptide sample was loaded onto Evotips.

Glycopeptide Enrichment

IMAC flow-through were desalted using C18 columns prior to glycopeptide enrichment. Glycopeptides were enriched using OASIS MAX solid-phase extraction cartridges. In brief, cartridges were sequentially conditioned with three washes of 1 mL acetonitrile, followed by three washes with 100 mM triethylammonium acetate buffer, three washes with water, and three washes with 95% acetonitrile containing 1% trifluoroacetic acid (TFA).

Desalted peptides in 95% acetonitrile containing 1% TFA were loaded to the cartridge twice to maximize glycopeptide binding. The cartridge was washed four times with 1 mL 95% acetonitrile containing 1% TFA, and the wash fractions were collected. Glycopeptides were then eluted with 50% acetonitrile containing 0.1% TFA, dried under vacuum, and reconstituted in 3% acetonitrile containing 0.1% formic acid for three injections. An aliquot corresponding to one LC-MS/MS injection was loaded onto Evotips for intact glycopeptide analysis on the Orbitrap Ascend Tribrid MS.

Analytical LC-MS Metabolomics Analysis and Stable Isotope Tracing

Chromatographic separation was performed on an Agilent 1290 Infinity II UHPLC system using a Waters Atlantis Premier BEH Z-HILIC VanGuard FIT column (1.7 mm, 2.1 mm x 100 mm). Mobile phase A was 20 mM NH_4CO_3, 5 µM medronic acid in water. Mobile phase B was 10% mobile phase A + 90% acetonitrile. The gradient profile was: 0 min, 95% B, 0.18 mL/min; 14 min, 55% B, 0.18 mL/min; 15 min, 20% B, 0.18 mL/min; 19 min, 20% B, 0.18 mL/min; 19.50 min, 95% B, 0.18 mL/min; 22 min, 95% B, 0.18 mL/min; 25 min, 95% B, 0.4 mL/min; stop time, 29 min. Column temperature was 40°C and autosampler temperature was 4°C. Injection volume was 3 µL. The LC system was coupled to a Bruker timsTOF-Pro MS equipped with an ionBooster electrospray ionization (ESI) source. The MS was operated in both positive and negative modes with tims on. The mass axis was calibrated at the beginning of every sample run. Data were acquired with Compass Hystar software (Bruker). The MS method is the default method of the 4D metabolomics method.

Nano LC-MS Metabolomics Analysis

Chromatographic separation was performed on an Thermo Fisher EASY-nLC 1200 system using an Aurora C18 column (25 cm x 75 µm, 1.7 µm). Mobile phase A was 0.1% formic acid in water. Mobile phase B was 0.1% formic acid in acetonitrile. The gradient profile was: 0 min, 1% B, 0.30 µL/min; 1 min, 1% B, 0.30 µL/min; 4 min, 35% B, 0.30 µL/min; 10 min, 70% B, 0.30 µL/min; 20 min, 95% B, 0.30 µL/min; 23 min, 95% B, 0.30 µL/min; 24 min, 1% B, 0.30 µL/min; stop time, 30 min, 1% B, 0.30 µL/min. Column temperature was 50°C and autosampler temperature was 4°C. Injection volume was 2 µL. The LC system was coupled to a Bruker timsTOF-HT MS. The MS was operated with tims on. Data were acquired with the Compass Hystar software (Bruker). The MS method is the default method of the 4D metabolomics method with stepping acquisition.

Nano LC-MS Lipidomics Analysis

Chromatographic separation was performed on an Thermo Fisher EASY-nLC 1200 system using an Aurora C18 column (25 cm x 75 µm, 1.7 µm). Mobile phase A was acetonitrile/water = 60/40 with 10 mM ammonium formate. Mobile phase B was isopropanol/acetonitrile = 90/10 with 10mM ammonium formate and 0.1 % formic acid. The gradient profile was: 0 min, 1% B, 0.25 µL/min; 1 min, 1% B, 0.25 µL/min; 3 min, 30% B, 0.25 µL/min; 6.5 min, 51% B, 0.25 µL/min; 11 min, 61% B, 0.25 µL/min; 15 min, 70% B, 0.25 µL/min; 20 min, 99% B, 0.25 µL/min; 25 min, 99% B, 0.25 µL/min; 26 min, 1% B, 0.30 µL/min; stop time, 30 min, 1% B, 0.30 µL/min. Column temperature was 50°C and autosampler temperature was 4°C. Injection volume was 2 µL. The LC system was coupled to a Bruker timsTOF-HT MS. The MS was operated with tims on. Data were acquired with the Compass Hystar software (Bruker). The MS method is the default method of the 4D lipidomics method with stepping acquisition.

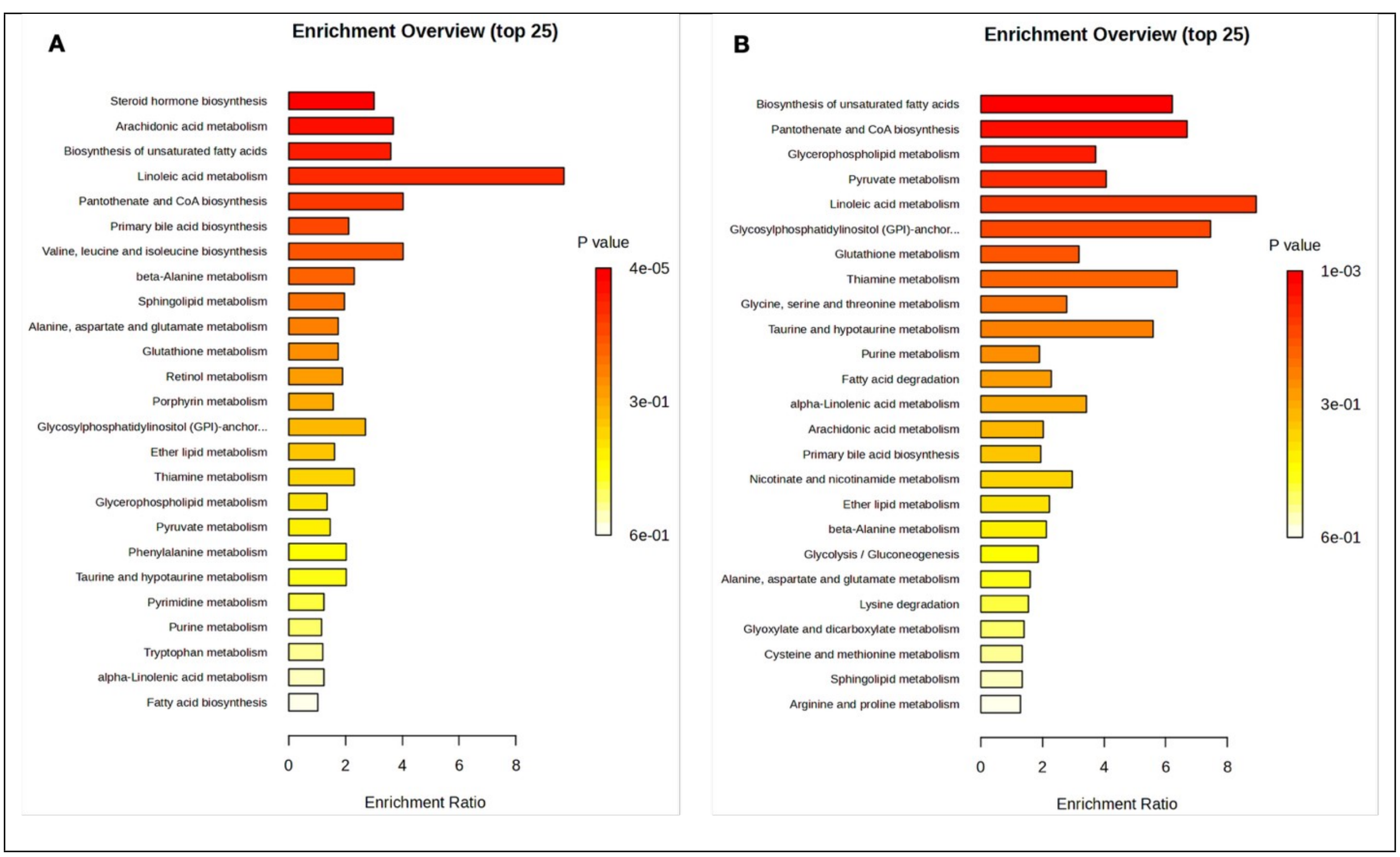

Fig. S1. MetaboAnalyst KEGG pathway enrichment analysis of HMDB-annotated features. (a) Pathway enrichment based on features detected by nLC-MS. (b) Pathway enrichment based on features commonly detected by both aLC-MS and nLC-MS.

Supplementary Figure
